# Health empowerment scripts: Simplifying social/green prescriptions

**DOI:** 10.3389/fpsyg.2022.889250

**Published:** 2022-11-03

**Authors:** Justin T. Lawson, Ross Wissing, Claire Henderson-Wilson, Tristan Snell, Timothy P. Chambers, Dominic G. McNeil, Sonia Nuttman

**Affiliations:** ^1^Health Nature Sustainability Research Group, School of Health and Social Development, Deakin University, Burwood, VIC, Australia; ^2^School of Architecture and Built Environment, Deakin University, Geelong, VIC, Australia; ^3^School of Psychology, Deakin University, Burwood, VIC, Australia; ^4^Institute of Health and Wellbeing, Federation University, Ballarat, VIC, Australia

**Keywords:** social prescriptions, green prescriptions, salutogenesis, health promotion, self-determination theory, green mind theory, health interventions, nature-based therapies

## Abstract

Social prescriptions are one term commonly used to describe non-pharmaceutical approaches to healthcare and are gaining popularity in the community, with evidence highlighting psychological benefits of reduced anxiety, depression and improved mood and physiological benefits of reduced risk of cardiovascular disease and reduced hypertension. The relationship between human health benefits and planetary health benefits is also noted. There are, however, numerous barriers, such as duration and frequencies to participate in activities, access, suitability, volition and a range of unpredictable variables (such as inclement weather, shifting interests and relocating home amongst others) impeding a comprehensive approach to their use on a wider scale. From a multidisciplinary perspective, this commentary incorporates a salutogenic and nature-based approach to health, we also provide a range of recommendations that can be undertaken at the patient level to assist in shifting the acknowledged systemic barriers currently occurring. These include using simple language to explain the purpose of health empowerment scripts, ensuing personal commitment to a minimum timeframe, enabling ease of access, co-designing a script program, providing ongoing motivational support and incorporating mindfulness to counter unexpected disruptions.

## Introduction

Health professionals [e.g. a general practitioner (GP) or psychologist] are increasingly offering non-medical interventions variously known as ‘social’ or ‘green’ prescriptions; which enable the health professional to collaborate with a link worker or community navigator who then facilitates a person-centred conversation to design the participant’s own solutions to well-being (Cook et al., 2019). These interventions can be undertaken in a variety of settings including nearby open space, urban parks, rugged wilderness, as well as virtual settings (Yu et al., 2018; Mackinnon et al., 2019; Horigome et al., 2020). The well-being conversation can prevent unnecessary GP attendance, reduce hospital emergency admissions, reduce social isolation and help support individuals with a range of conditions (Chatterjee et al., 2017). In Australia, 14% of patients report receiving social prescribing, and of these, 91% reported it was helpful (Friends for Good, 2021). There are several known factors that demonstrate a strong need for viable, sustainable, complementary approaches (Ananthapavan et al., 2021). These include rising costs in healthcare, most recently highlighted in media with about 30 to 40% of Australian GPs having switched to mixed or private billing within the last 12 months (Opray, 2022), environmental costs in pharmaceuticals in the context of resource extraction and carbon footprint, with the pharmaceutical industry emitting more than the automotive industry (Belkhir and Elmeligi, 2019) and public health messaging of spending time outdoors to counteract the increasing amounts of time indoors (further exacerbated by covid-19 lockdown measures). In contrast to medical interventions, however, it is unclear what evidence is used to inform decision-making and whether social prescriptions are tailored for individual patients. Emerging grey literature highlights the support that health professionals are providing for the intervention (Jorgensen and Robinson, 2020; National Health Service, 2020; Broom, 2022; Green Adelaide, 2022), despite hurdles in motivation (Nix, 2022; Ryan, 2022).

Husk et al. (2019) note that research on social prescribing is limited, low quality and likely to be biased, whereas other researchers have commented that current research is insufficient to determine success or value for money (Bickerdike et al., 2017). Although social prescribing may reduce demand on health services, this is only amongst patients who follow recommendations (Dayson and Bashir, 2014). One plausible reason for low uptake in patients may be due to a lack of tailoring of social prescriptions to patient needs and temperament. Further limitations to uptake may be due to undeveloped training and education in social prescriptions, with little evaluation frameworks to support education, limited opportunities for collaboration between health and community sectors, as well as the lack of funding at various levels of government (Royal Australian College of General Practitioners, 2020). Hence, the aim of this paper is to review the diverse types of social prescriptions and provide recommendations that may support health practitioners to design personalised social prescriptions that best suit the person seeking treatment, with a focus on nature-based treatments and settings.

Following current trends in systems thinking for health that draws on multi-, inter- and transdisciplinary approaches (de Savigny et al., 2017), the commentary on social prescriptions is informed by researchers in psychology, exercise sciences, health sciences, environmental health, food systems, sustainability, landscape architecture and education. We hope this can provide a fair starting point to open further discussions and inputs from other disciplines and practitioners to provide a comprehensive and inclusive approach to appropriate healthcare with respect to social prescriptions.

Despite criticisms of social prescriptions, there is a concerted effort amongst health professionals to explore and address the origins of health, as highlighted by Kickbusch (1996). This effort, or approach, is best encapsulated by the earlier work of Antonovsky (1979) who developed the term “salutogenesis.” Conceptualised as a stress-oriented approach to human health, salutogenesis focuses on individual resources that improve and maintain progression towards health (Lindström and Eriksson, 2005). In practice, life experiences help shape one’s sense of coherence (a global orientation); life is understood as more or less comprehensible, meaningful and manageable (Mittelmark and Bauer, 2017). Whilst pathogenesis focuses on factors that cause disease, salutogenesis focuses on factors that support human health and well-being (Antonovsky, 1979). Thus, there are opportunities in fostering resilience and a sense of purpose amongst individuals and communities by promoting a salutogenic approach (Cook et al., 2019). By developing skills, endorsing attributes and supporting locally based resources, a sense of coherence between health and illness can be provided (Cook et al., 2019).

In supporting the salutogenic approach and the various disciplines that are represented in the authorship of this commentary, numerous theories, models and theoretical frameworks have been considered. We acknowledge that there are many available to assist in framing the overall directive of social prescriptions. We also note that this may have influenced the uptake of social prescriptions, considering the wide range of health practitioners involved (noting Tierney et al.’s identification of 75 title descriptions for care navigators (Tierney et al., 2019). One of the key models considered is Bronfenbrenner’s ecological systems theory model where micro-meso-macro settings influence the development of a person (Bronfenbrenner, 1977). Fox’s ethics and the built environment (Fox, 2000) provides a macro framework that incorporates five capitals of natural, human, social, economic and built and numerous meso theories and models as well, highlighting the vast range of approaches that individuals have with (predominantly) residential landscapes. Situated within ethics and the built environment is the self-determination theory (SDT; Deci and Ryan, 1985), explaining motivations based on autonomy, competence and relatedness with extrinsic and intrinsic outcomes. Further to these considerations is the revised mandala of health (Langmaid et al., 2020), which posits a deep relationship with nature that is sympathetic and empathetic, fully cognisant of the ecosystem services and the full human experience inclusive of body, mind and spirit. Whilst the mandala draws parallels with Bronfenbrenner’s bioecological model, it also reflects the green mind theory (GMT) proposed by Pretty et al. (2017), as individuals experience a ‘green mind’ through heightened neuro-physiological functioning during green space exposure. This increased physiological activation, in combination with strengthened motives towards nature, help develop the connection between micro-meso-macro settings (Pretty et al. 2017). Considering the far-reaching application of Pretty et al.’s 10-point action plan (2017; p. 12) that addresses lifestyle and behaviour change as well as changes in infrastructure, policies and resourcing, we feel that SDT and GMT best underpins the application of social prescriptions. Certainly, SDT is gaining traction in a variety of settings and disciplines that also acknowledges the importance of the natural environment (Center for Self-Determination Theory, 2022).

More recently, the World Health Organization has released the Geneva Charter for Well-Being (World Health Organization, 2021). Consisting of five areas firmly addressing planetary health, universal health coverage and digital technology, it promotes health in a salutogenic framework, explicitly stating “that people and communities are enabled to take control of their health and lead fulfilling lives with a sense of meaning and purpose, in harmony with nature, through education, culturally relevant health literacy, meaningful empowerment and engagement” (World Health Organization, 2021; p. 4). Thus, health empowerment scripts with a focus on nature-based activities are well aligned to overarching principles.

Currently, whilst there is considerable interest in the community and support for a variety of options of social prescribing (Royal Australian College of General Practitioners, 2020), there is little information on standards of procedure, types of appropriate activities and wide conflation of terms. As such, this paper endeavours to establish a set of recommendations for treatments to assist in the decision-making processes for health practitioners to ensure that the appropriate type of activities is employed for the person seeking treatment.

## Salutogenics and social prescriptions – Types of activities

Salutogenic approaches cover many dimensions and conditions, and there is a significant conflation of terms that are currently in use. One that is used considerably and in a variety of settings is ‘social prescription’. Approaches to social prescribing range from long-term condition management to volunteer opportunities with a focus on well-being through diverse activities, which can include group interactions or individual exercises that can be held in natural or indoor settings (Coopes, 2020). This demonstrates that there are considerable vagaries for the term ‘social prescription’, consequently providing an opportunity for refinement and clarity to ensure that appropriate interventions are used. Social prescribing could provide a valuable addition to the existing range of healthcare options in Australia. However, to date, the adoption of social prescribing as an organised program of support has been limited, in part due to a perceived lack of objective evidence, limited time for GPs and limited understanding of suitable places to undertake outdoor activities (Royal Australian College of General Practitioners, 2020; Zurynski et al., 2020). Whilst we outline the differences of the various terms used in different disciplines, we also seek to identify the similarities as a way of enhancing clarity.

We therefore categorise interventions based on activity and setting. Activities can be either passive or active and undertaken in (a) solitary situations, (b) individually, but with shared exchanges with others and (c) as a collective, group exercise. These activities are then pursued in two settings: (I) indoor and (II) outdoor. Indoor settings can be a private, personal space (e.g. one’s home) or shared (e.g. a workplace, library, café, gym, etc.), and the outdoor setting can again be private (e.g. one’s backyard) or shared (e.g. the local park, nature reserve, beach, etc.). An adjunct to the activity- and setting-based intervention is the use of augmented and/or virtual reality (AR/VR) devices. These devices can be used as supplements to usual activities and settings. Considering the recent developments of Meta (Nix, 2022; Reuters, 2022; Wolpin, 2022), and its promotion as a setting where innumerable activities can occur, there is another layer to consider how social prescriptions can be deployed.

Parallels to a salutogenic approach to well-being currently exist amongst practicing mental health professional who use formulation to design therapeutic interventions. Formulation draws upon psychological theory to allow practitioners to make a hypothesis about a person’s difficulties based on psychological theory (Johnstone and Dallos, 2014). Formulations are co-constructed with the patient. These hypotheses typically include explanations about the underlying causes, precipitating issues and maintaining factors associated with presenting difficulties. As a result, therapies can then be designed to identify key targets and interventions for change that address not only the presenting symptoms, but also the underlying causes and maintaining factors of these symptoms (Johnstone and Dallos, 2014).

Some authors have proposed specific models that apply formulation to behavioural interventions, interventions that recommend behavioural actions designed to improve health, which are the closest comparison to a health empowerment script. Haynes and Williams (2003) argue that for individuals with multiple behavioural problems, these problems can interact in multiple and causal ways, and hence, it is important to consider what underlying factors have the biggest impact on presenting issues so that the most effective interventions can be selected. Using a ‘functional analytic’ approach, the authors propose estimating the impact, relationships, causal paths and modifiability of various behaviours, to design interventions that have the largest effect. Hence, an approach to health empowerment scripts might benefit from drawing on a formulation approach such as this, so that interventions are individually designed, co-constructed with the patient and carefully considered to have the most significant impact on the underlying causes of health problems.

## Art to wilderness – Terms of social and green prescriptions

The range of terms employed for social prescriptions are wide and varied, dependent on the activity-setting as discussed earlier. Husk et al. (2020) also note that “‘social prescribing’ is not a single intervention but a pathway and series of relationships, all of which need to function to meet patient needs” (Husk et al., 2020; p. 309). Table 1 below classifies the various labels with associated interventions and examples, which have been sourced from Jepson et al. (2010), Bragg and Leck (2017), Chatterjee et al. (2017), Robinson and Breed (2019) and Patel et al. (2021). With the examples provided, interventions can be a suite of offerings, again dependent on the interests and capabilities of the individual. Incorporating formulations will further help in determining the specific activities linked to the examples provided; for example, music, being an ‘arts on prescription’ intervention, can be further refined as an individual (e.g. learning to play an instrument), or collective (e.g. participating in a choir) activity.

**Table 1:** Social and green prescription labels, intervention types and examples.

Label	Intervention type	Examples
Social Prescription	Arts on Prescription	Painting/drawing
		Crafts-sculpture, weaving
		Dance
		Drama
		Music
		Writing/Journaling
	Books on Prescription	Self-help books
		Reading for leisure
		Book club
	Education on Prescription	Money management
		Cooking
		Organisational Skills
		Language learning
		Other courses
	Exercise Referral/Exercise on Prescription	Gym
		Yoga
		Swimming
	Green Prescription (note that this intervention is also a label in itself)	Walking in park
		Gardening
		Community Gardening
		Visiting beach
		Visiting national park
	Healthy Living Initiatives	Healthy eating program
		Stop smoking program
		Alcoholics Anonymous
		Narcotics Anonymous
	Signposting/Information Referral	Financial advice
		Housing support
Supported Referral	Examples as above and below
Green Prescription, Nature Prescription, Nature-Based Prescription, Park (Rx) Prescription	Physical/mental activity in natural setting, Outdoor education, Outdoor adventure (or OA therapy), Bush adventure (or BA therapy), Wilderness experience, Wilderness adventure (or WA therapy)	Bushwalking
		Hiking
		Trail-riding
		Rock-climbing
		Mountaineering
		Abseiling
		Orienteering
		Wildlife watching/safari
		Surfing
		Rafting
		Sailing
		Swimming
		Scuba diving
		Water skiing
		Alpine skiing
		Camping
		Caving
		Fishing
		Hunting
		Horse/camel riding
		Skydiving		
		Parasailing
		Paragliding
		Hang gliding
	(Natural) Forest therapy (*shinrinyoku*), aka forest bathing	Bushwalking
	Horticultural therapy, aka therapeutic horticulture, social horticulture, gardening therapy	Home gardening
		Community gardening
	Animal Assisted Therapy	Watching animals
		Petting animals
		Handling animals
		Walking with animals
		Riding with animals	Care farming	Handling animals

## Benefits

Whilst benefits of social prescriptions with interventions focussing on creative arts, education and exercise include improvements on measures of quality of life, mental and physical health, well-being, healthy behaviours and social engagement (Pilkington et al., 2017; Pescheny et al., 2020), overall, results are mixed (Pescheny et al., 2020; Zurynski et al., 2020). The diversity of outcome measures and study designs have inadvertently muddied the waters, as well as the vast differences in models of delivery (for instance, the number of sessions, duration of support and types of workforce involved). As such, meta-analyses of such programs are problematic (Pescheny et al., 2020).

Much, however, has been stated about the benefits of contact with nature, with many recent meta-analyses demonstrating the psychological (e.g. reduced anxiety, depression, improved mood) and physical (e.g. reduced risk of cardiovascular disease, reduced hypertension) benefits of human contact with nature (Frumkin, 2003; Bowler et al., 2010; Hartig et al., 2014; Kondo et al., 2020; Roberts et al., 2021). Further, there are benefits for the natural environment, when actions undertaken individually and collectively can improve the functioning of the planet (i.e. reduction of greenhouse gas emissions through behaviour change, or improved biodiverse habitats through individual and community participation programs, etc.; Robinson and Breed, 2019). Thus, there is also the opportunity to inculcate an approach that is sustainable on several fronts, and in so doing create co-benefits.

## Barriers

Whilst the benefits of social prescriptions have been well reported (Chatterjee et al., 2017; Pilkington et al., 2017; Pescheny et al., 2020; Zurynski et al., 2020; Patrick et al., 2021), there are still numerous barriers that make it difficult for health practitioners to provide comprehensive and effective plans for their patients to follow. Zurynski et al. (2020) highlight barriers for GPs and other health professionals, link-workers and community organisations, patients, as well as across various systems (i.e. knowledge, health, governance). Acknowledging that many of the barriers outlined in previous research will take considerable coordination and funding from multiple sources to resolve (and thus potentially an inordinate amount of time), we have chosen to focus on more manageable patient-centred barriers that we consider to be not insurmountable. By tackling these as a starting point, it is hoped that the more systemic barriers consequently can be addressed.

### Time

Evidence testing the prominent restoration/recovery therapies, such as Attention Restoration Therapy (ART) and Stress Reduction Therapy (SRT), suggest that benefits are evident from as little as 40 s of exposure, up to 55 min (Berto, 2005; Berman et al., 2008; Lin et al., 2014; Lee et al., 2015; Pilotti et al., 2015; Chen et al., 2020). These have been conducted as singular events with one or two repetitions. Other studies have been conducted over longer periods, e.g. three, 30-min outdoor walks each week for 2 weeks (Duvall, 2011, 2013) and 90 min per week for 5 weeks (Lymeus et al., 2018).

Duration is also dependent on the intensity of the natural environment (Shanahan et al., 2016) and the activity undertaken (e.g. reading in nature vs. exercise). For example, regarding ‘green exercise’, Barton and Pretty (2010) highlight activities that are light in intensity and for a brief period (5 min) provide improved self-esteem and mood. As the activity increases in intensity and duration (up to half a day), esteem and mood decreases, yet with a full day of activity there is an improvement in self-esteem and mood but not to the same levels as the short duration. This raises questions about the very nature of the activities, as reading a book for 5 min will more than likely not have the same impact as a quick five-minute stroll around a park. Nevertheless, an activity being undertaken at a singular point of time (e.g. a day trip to a park) will provide some immediate relief but will not be sustained.

### Access

In addition, an activity based on a hard to get to location or requiring multiple modes of transport (i.e. low-level access) will achieve some success but again will not be a sustainable approach. The cost of the activity also affects accessibility; ideally, the activity needs to be free or along a sliding scale of affordability dependent on the patient’s current income status.

### Suitability

Suitability has some similarities with access; the location may have some existing structural barriers (e.g. steps, gravel paths or uneven surfaces, lack of handrails, shade or seating, etc.), however, there are other considerations in relation to the patient. For example, they may have some pre-existing conditions that could preclude them from accessing the outdoor setting or social gathering. A patient’s physical fitness, as well as their mental and social aptitude, aligning with their personal interests, will have significant influences on their approach to utilising health empowerment scripts.

### Volition

Of the current reviews in social prescriptions (Chatterjee et al., 2017; Pescheny et al., 2020; Zurynski et al., 2020), none have mentioned the volition or independence of the patient in uptake of social prescriptions. Granted, the activities are prescribed after consultations and with needs assessments being undertaken. Patients are often unaware of the diverse services provided and link workers/care navigators are vital agents in providing the appropriate information to those patients. Ultimately, the intervention needs not to be ‘prescribed’ *per se*, rather it needs to be an easily adopted suite of options, where the individual can make informed decisions towards a fully independent, ‘empowered’ status.

### Variables

Further complexities to uptake of scripts include a range of unknowns, such as goals not being reached in certain timeframes, shifting interests, changes in living conditions, disruptive weather, traffic conditions or social distancing requirements. Some of these may disrupt the continuity briefly, whereas others may be a permanent ending of the service. When a patient’s physical or mental health is already compromised, it may be difficult to obtain the levels of commitment required to ensure the full benefit of the service being used.

By overcoming these barriers, with strategies to handle unknown variables, the empowered individual, who has the ease of access to services, can sustain the practice over the course of their life. This supports the findings of Husk et al. (2020) regarding patients’ views of social prescriptions being of benefit, presented in an acceptable way that matches their needs and expectations, and if the activity is both accessible with supported transport. By becoming part of their daily or weekly routine, the habituated activity has the potential to provide other benefits, especially if located within natural settings. This speaks to the term ‘green prescriptions’ where nature-based activities can potentially inculcate nature-supportive endeavours, such as gardening, bush care, wildlife monitoring, etc. This highlights the issue of terminology employed for social prescriptions, as discussed earlier.

## Recommendations

SDT and GMT are key theories that support the salutogenic approach to health empowerment scripts, with Bhatti et al. (2021) and Pretty et al. (2017) providing evidence of strong adherence based on meaningful outcomes and time spent on activities. Recognising that there are barriers at institutional and policy level, we provide a range of feasible recommendations at the individual level that will support health practitioners in guiding patients to appropriate programs.

1. Using simple language to explain to the patients the purpose of health empowerment scripts. Bertotti and Frostick (2018) identified that study participants had no idea of the social prescription model despite the referrals from their GP, whereas Fixsen et al. (2020) found that lack of prior information and GP involvement also affected patient uptake.
2. Ensuring that individuals can commit to a certain amount of time to undertake the program. To affect changes in behaviour, Pretty et al. (2017) recommend a minimum of 50 days at 1 h per day, or 100 days (approximately 3 months) at half an hour per day.
3. Enabling ease of access for patients. Whilst there may be considerable structural barriers already in place, ensuring that patients can be supported either with readily available transport or financially will result in long term adherence. Drinkwater et al. (2019) highlight the importance of building social capital in local areas done by link workers.
4. Co-producing an action plan with the patient is critical for supporting SDT and the salutogenic approach, which enables a sense of suitability of the activity. Co-design approaches have been found to support positive health and well-being outcomes (Chesterman and Bray, 2018; Drinkwater et al., 2019; Thomas et al., 2021).
5. Ongoing motivational support, as well as determining what is comfortable for the patient will bring about the optimum outcome, with the intention that the patient can be empowered to undertake the chosen activity with little supervision. Hassan et al. (2020) found that study participants from disadvantaged backgrounds were determined, having access to services, to further their self-development and independence.
6. Incorporating mindfulness throughout to counter the effects of unexpected disruptions. Mindfulness itself can be viewed as a prescription (Pretty et al., 2017), yet is also required to check the continuity of the activities undertaken. Knowing that not all things go to plan and allowing some flexibility to access will ensure better uptake by patients (Moffatt et al., 2017; Robinson and Breed, 2019).

## Conclusion

To conclude, whilst there is a strong appetite for social and green prescriptions to be incorporated as a health intervention at a community level, there is still considerable work needed to be undertaken to ensure that they are supported amongst health professionals and various government agencies. By providing a categorisation of terms and a set of recommendations at the patient level, it is hoped that the systemic barriers can be shifted such that there is further uptake of a valuable intervention in the future. We feel that health empowerment scripts, with a focus on nature-based activities, are a tool to fully support the Geneva Charter’s focus on “meaningful empowerment and engagement.”

## Data availability statement

The original contributions presented in the study are included in the article/supplementary material, further inquiries can be directed to the corresponding author.

## Author contributions

JTL, RW, and CH-W conceived the idea of Health Empowerment scripts. All authors contributed to the article and approved the submitted version.

## Conflict of interest

The authors declare that the research was conducted in the absence of any commercial or financial relationships that could be construed as a potential conflict of interest.

## Publisher’s note

All claims expressed in this article are solely those of the authors and do not necessarily represent those of their affiliated organizations, or those of the publisher, the editors and the reviewers. Any product that may be evaluated in this article, or claim that may be made by its manufacturer, is not guaranteed or endorsed by the publisher.
